# Supplementary material for: Time trend analysis of perinatal mortality, stillbirth, and early neonatal mortality of multiple pregnancies for each gestational week from the year 2000 to 2019: A population-based study in Japan
Source: PLoS One. 2022 Jul 25;17(7):e0272075. doi: 10.1371/journal.pone.0272075 (PMC9312402; doi:10.1371/journal.pone.0272075)
Supplement: Attachment — Submitted filename: Responses to journal requirements.docx [file pone.0272075.s007.docx]

Responses to journal requirements

We would like to thank you for your critical comments and insightful suggestions, which have helped us improve our manuscript. As indicated in the responses below, we have integrated all your comments and suggestions in the revised version of our manuscript.

[Comment]

1. Please ensure that your manuscript meets PLOS ONE's style requirements, including those for file naming.

[Response]

We have reedited the manuscript format according to the style stipulated by PLOS ONE.

[Comment]

2. PLOS requires an ORCID iD for the corresponding author in Editorial Manager on papers submitted after December 6th, 2016. Please ensure that you have an ORCID iD and that it is validated in Editorial Manager.

[Response]

We have registered the ORCID iD of the corresponding author in the Editorial Manager.

The ID is: Masahiko Nakata 0000-0002-3621-1251

[Comment]

3. Please include captions for your Supporting Information files at the end of your manuscript, and update any in-text citations to match accordingly.

[Response]

We have added the supporting information with captions at the end of the manuscript.

Additionally, we have updated the in-text citations referring to the supporting information in the text.
